# Involvement of Progranulin and Granulin Expression in Inflammatory Responses after Cerebral Ischemia

**DOI:** 10.3390/ijms20205210

**Published:** 2019-10-21

**Authors:** Ichiro Horinokita, Hideki Hayashi, Rika Oteki, Risa Mizumura, Tatsuaki Yamaguchi, Akane Usui, Bo Yuan, Norio Takagi

**Affiliations:** 1 Department of Applied Biochemistry, Tokyo University of Pharmacy and Life Sciences; Hachioji 192-0392, Japan; y101156@toyaku.ac.jp (I.H.); hhayashi@toyaku.ac.jp (H.H.); y144039@toyaku.ac.jp (R.O.); risa.piku.0811@gmail.com (R.M.); coupy.is.all.lead@gmail.com (T.Y.); akane_1819@yahoo.co.jp (A.U.); 2 Laboratory of Pharmacology, School of Pharmacy, Faculty of Pharmacy and Pharmaceutical Sciences, Josai University, Sakado 350-0295, Japan; yuanbo@josai.ac.jp

**Keywords:** cerebral ischemia, progranulin, granulin, elastase, inflammatory cytokine, microglia

## Abstract

Progranulin (PGRN) plays a crucial role in diverse biological processes, including cell proliferation and embryonic development. PGRN can be cleaved by neutrophil elastase to release granulin (GRN). PGRN has been found to inhibit inflammation. Whereas, GRN plays a role as a pro-inflammatory factor. However, the pathophysiological roles of PGRN and GRN, at early stages after cerebral ischemia, have not yet been fully understood. The aim of this study was to obtain further insight into the pathologic roles of PGRN and GRN. We demonstrated that the amount of PGRN was significantly increased in microglial cells after cerebral ischemia in rats and that neutrophil elastase activity was also increased at an early stage after cerebral ischemia, resulting in the production of GRN. The inhibition of neutrophil elastase activity suppressed PGRN cleavage and GRN production, as well as the increase in pro-inflammatory cytokines, after cerebral ischemia. The administration of an elastase inhibitor decreased the number of injured cells and improved the neurological deficits test scores. Our findings suggest that an increase in the activity of elastase to cleave PGRN, and to produce GRN, was involved in an inflammatory response at the early stages after cerebral ischemia, and that inhibition of elastase activity could suppress the progression of cerebral ischemic injury.

## 1. Introduction

Ischemic stroke continues to be a leading cause of disability and mortality worldwide. One of the therapeutic agents for stroke is tissue plasminogen activator (t-PA) as a clinical application. Although, the clinical benefits of t-PA treatment manifest when it is administered within 4.5 h of stroke onset, delayed treatment with t-PA leads to severe complications. This is a result of increased risk of hemorrhagic complications, a breakdown of the blood-brain barrier (BBB), and the neurotoxic effects of t-PA [1,2]. The increase in vascular permeability and disruption of the BBB, caused by cerebral ischemia, are due to pathological consequences and can be the initiating factors for the development of cerebral infarctions. It has been shown that macrophages and neutrophils, infiltrating through an injured-BBB, contribute to the release of inflammatory cytokines in the brain parenchyma. These released inflammatory cytokines exacerbate inflammatory responses, provoke neuronal cell death, and also affect neurogenesis [3,4]. Therefore, controlling inflammation-related factors in the infarct area and its surrounding area can be a link to the development of new therapeutic strategies against ischemic stroke.

Progranulin (PGRN) is a cysteine-rich protein that is crucial in diverse biological processes, such as cell proliferation and embryonic development [5] and is known to perform various biological functions, such as protection against vascular disorders and neuronal disorders [6,7,8]. PGRN can be subjected to protease-mediated cleavage by enzymes, such as neutrophil elastase [9] to release granulin (GRN) [10]. Both PGRN and GRN have biological activities, although often with those opposing functions in immunoreactions. In a mouse model of rheumatoid arthritis, PGRN was found to inhibit inflammation by suppressing the TNF-α-mediated intracellular signaling pathway [11]. Also, PGRN is a neurotrophic factor that promotes cellular survival and stimulates neurite outgrowth in the nervous system [12]. Therefore, PGRN is suggested to be a promising neuroprotection option that helps to improve the neurological functions after ischemia, which is thought to be mostly due to its role in decreasing the expression of inflammatory cytokines and increasing that of anti-inflammatory cytokines [9,11]. On the other hand, GRN plays a role as a pro-inflammatory factor. It has been reported that A549 and SW-13 cells produce large amounts of IL-8 in response to the addition of recombinant GRN to the culture medium [9]. These findings raise the possibility that PGRN and GRN play a pivotal role in inflammatory responses during the acute phase of brain ischemia. However, the pathophysiological roles of PGRN and GRN, after cerebral ischemia, are not yet fully understood. Therefore, the aim of this study was to obtain further insight into the pathologic roles of PGRN and GRN. In the present study, we used the microsphere-induced cerebral embolism (ME) model in rats. ME induces widespread formation of small emboli and multiple infarct areas in the brain. Previous studies demonstrated that scattered necrotic areas, variable in size and shape, were seen mainly in the parietotemporal cortex, corpus callosum, hippocampus, thalamus, and lenticular nucleus of the ipsilateral hemisphere after ME [13,14]. Thus, this model is considered to mimic focal ischemia-induced human stroke [15] or multi-infarct dementia [16]. Using an in vivo ischemic animal model, we examined pathophysiological alterations of PGRN and GRN after cerebral ischemia and also the effects of an elastase inhibitor on cell injuries and neurological deficits at early stages after cerebral ischemia.

## 2. Results

### 2.1. Time Course of Changes in the Level and Cellular Source of PGRN after Cerebral Ischemia

**Figure 1:**
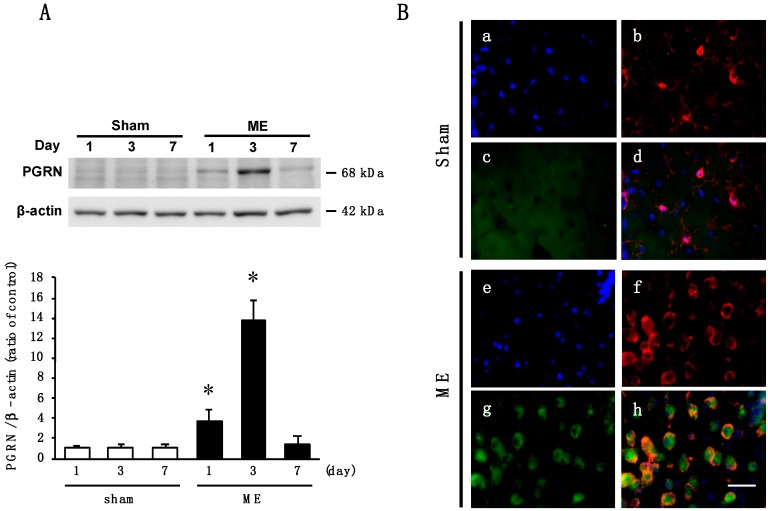
(**A**) Time course of changes in the levels of Progranulin (PGRN) protein in the sham- (sham; white bars) and microsphere-induced cerebral embolism (ME)-operated groups (ME; black bars) on days 1, 3, and 7 after surgery. Bands corresponding to PGRN were scanned, and the scanned bands were normalized by β-actin on the same blot. Results are expressed as the mean ratio of non-operated (control) group ± SD (*n* = 9 each). * Significant difference from the sham-operated group (*p* < 0.05); (**B**) Cellular localization of PGRN in the cortex of sham-operated (a–d) and ME-operated (e–h) rats on day 3 after surgery. Images of triple staining (merge, d and h) with Hoechst 33342 (blue, a and e), Iba-1 (red, b and f), and PGRN (green, c and g). The scale bar represents 30 μm.

We first examined the time course of changes in the levels of PGRN proteins in the cerebral cortex after ME. The level of PGRN protein of ME-operated rats was significantly increased as compared with that of sham-operated rats on day 3. Then, ME-operated rats decreased to the same level as the sham-operated ones on day 7 after the operation (Figure 1A). As the amount of PGRN protein was significantly increased on day 3 after. 

ME, we next used the immunohistochemical analysis to examine the localization of PGRN protein in the cortex of sham- and ME-operated rats on day 3 after the operation. PGRN was not detected in Iba-1-positive microglia in the sham-operated rats (Figure 1B). In our preliminary experiments, glial fibrillary acidic protein (GFAP)-positive astrocytes in sham-operated animals did not express PGRN, although PGRN was detected in MAP2-positive neuron. Iba-1-positive microglia from the ME-operated rats expressed PGRN (Figure 1B). Iba-1-positive microglia were dispersed with a ramified form in the sham-operated rats, whereas round and ameboid-like microglia expressing PGRN were evident after ME (Figure 1B). The number of PGRN-positive microglia was increased on day 3, after ME, relative to that of sham-operated rats (Figure 1B).

### 2.2. Effect of Oxygen/Glucose Deprivation (OGD) on mRNA Levels of PGRN, TNF-α, and IL-1β in Cultured Microglia

**Figure 2:**
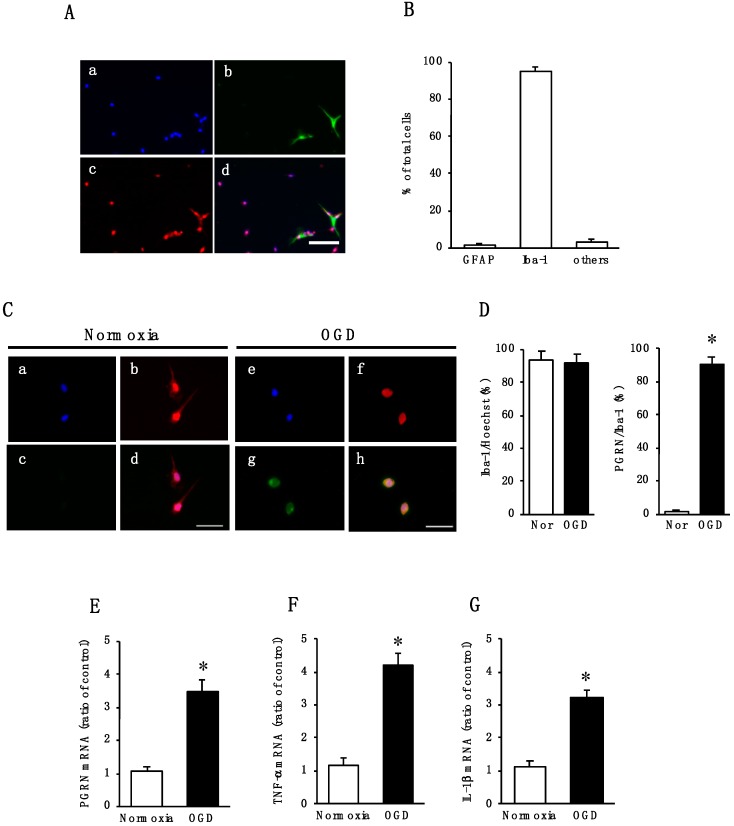
(**A**) Cortical cells fixed at 20 days in vitro and triple stained (merge, d) with glial fibrillary acidic protein (GFAP) (green, b) and Iba-1 (red, c), and Hoechst 33342 (blue, a). The scale bar represents 100 μm; (**B**) the numbers of GFAP- and Iba-1-positive cells were counted. The results are expressed as the percentage of these cells among the total number of Hoechst-positive cells and as the means ± SD (*n* = 7 independent experiments); (**C**) images of triple staining (merge, d and h) with Hoechst 33342 (blue, a and e), Iba-1 (red, b and f), and PGRN (green, c and g) under Normoxia (a–d) or OGD (e–h) treatment. The scale bar represents 30 μm; (**D**) the numbers of Iba-1- and PGRN-positive cells under normoxia (Nor) or OGD were counted. Five images were made per experiment, and 26–100 cells were counted per image, and the average of 5 images per experiment was calculated. The results are expressed as the percentage of these cells among the total number of Hoechst-positive cells (Iba-1/Hoechst) and that of Iba-1-positive cells (PGRN/Iba-1), and as the means ± SD (*n* = 9 independent experiments; the total number of Hoechst-positive cells counted: 2223 under normoxia and 2244 under OGD). * Significant difference from the normoxic group (*p* < 0.05); (**E**) Effects of Normoxia or OGD exposure on the expression of PGRN mRNA in microglia. The results are expressed as the mean ratio of the Normoxia or OGD to the control group ± SD (*n* = 8 independent experiments). * Significant difference from the normoxic group (*p* < 0.05); (**F**,**G**) Effects of Normoxia or OGD exposure on the expression levels of TNF-α (**F**) and IL-1β (**G**) mRNA in microglia. The results are expressed as the mean ratio of the Normoxia or OGD to the control group ± SD (*n* = 8 independent experiments). * Significant difference from the normoxic group (*p* < 0.05).

We next focused on PGRN expressed in microglia. To examine the expression of PGRN, we used cultured cortical microglia. As demonstrated by immunocytochemical analysis, glial cultures contained predominantly microglia (Figure 2A): 95.4  ±  2.3% Iba-1-positive microglia, 1.2  ±  0.9% GFAP-positive astrocytes, and 3.5  ±  1.5% other cells (Figure 2B). The subsequent experiments were performed by using these cultured cells as microglial cells.

We examined whether changes in the expression of PGRN occurred in primary cultures of microglia after OGD treatment, which is related to the pathogenesis of cerebral ischemia. There were no changes in the number of Iba-1-positive cells between normoxia and OGD condition (Figure 2C,D). At 24 h of normoxic treatment, PGRN proteins were not detected in microglia (Figure 2C,D). On the other hand, Iba-1-positive microglial cells expressed PGRN protein at 24 h of OGD treatment (Figure 2C,D). Furthermore, the expression of PGRN mRNA was significantly increased in microglial cells at 24 h of OGD treatment (Figure 2E). As it has been suggested that PGRN is linked to inflammatory responses, we also determined the expression of pro-inflammatory cytokines TNF-α and IL-1β mRNAs in the microglia. OGD treatment significantly increased the mRNA levels of TNF-α (Figure 2F) and IL-1β (Figure 2G) compared to those under normoxia. 

### 2.3. Time Course of Changes in Activity of Elastase and the Level of GRN after Cerebral Ischemia

**Figure 3:**
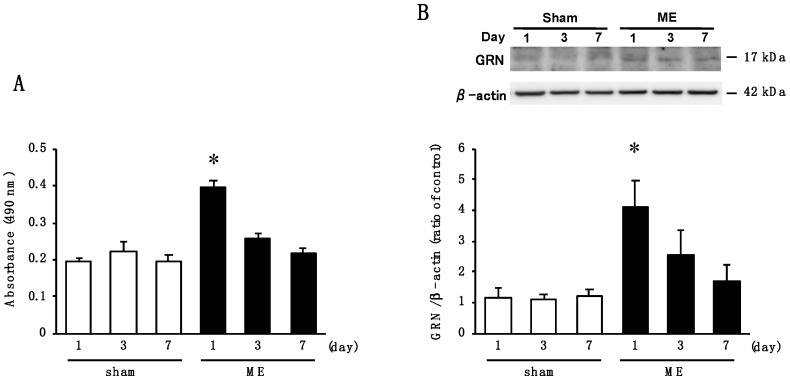
(**A**) Time course of changes in the tissue elastase activity in the sham-operated (sham; white bars) and ME-operated groups (ME; black bars) on days 1, 3, and 7 after surgery. The results are expressed as the mean ± SD (*n* = 6 each). * Significant difference from the sham-operated group (*p* < 0.05); (**B**) Time course of changes in the levels of granulin (GRN) protein in the sham-operated (sham; white bars) and ME-operated groups (ME; black bars) on days 1, 3, and 7 after ME. Bands corresponding to GRN were scanned, and the scanned bands were normalized by β-actin on the same blot. Results are expressed as the mean ratio of the non-operated (control) group ± SD (*n* = 8 each). * Significant difference from the sham-operated group (*p* < 0.05).

PGRN can be subjected to protease-mediated cleavage by neutrophil elastase [9] to release peptides called GRN [10]. Therefore, we examined whether changes in elastase activity was associated with GRN production in the brain at early stage after cerebral ischemia. Elastase activity on day 1 after ME was significantly increased compared to the sham-operated rats (Figure 3A). However, there were no significant differences in enzymatic activities between sham- and ME-operated rats on days 3 and 7 (Figure 3A). Therefore, we next examined the time course of changes in the levels of GRN in the cerebral cortex after ME. The amount of GRN protein was significantly increased on day 1 after ME compared with that for the sham-operated rats (Figure 3B).

### 2.4. Time Course of Changes in mRNA Levels of TNF−α, IL-1β, IL-10, and TGF-β after Cerebral Ischemia

**Figure 4:**
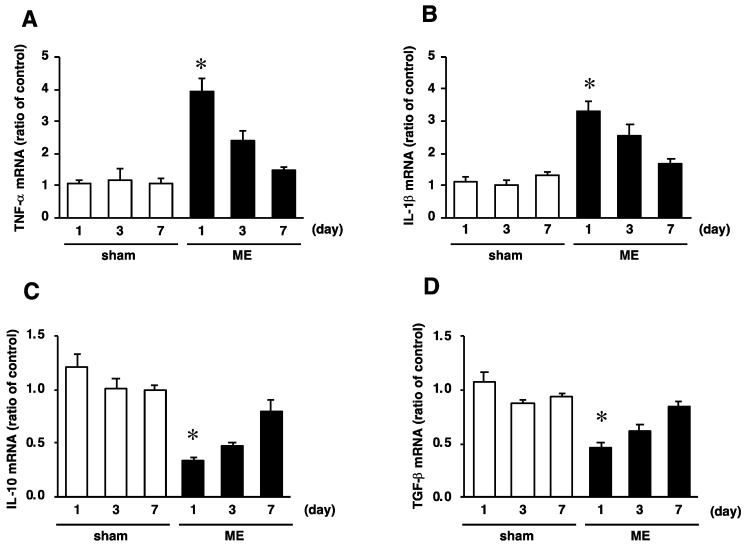
Time course of changes in the mRNA levels of TNF-α (**A**), IL-1β (**B**), IL-10 (**C**), and TGF-β (**D**) in the sham-operated (sham; white bars) and ME-operated groups (ME; black bars) on days 1, 3, and 7 after surgery. Results are expressed as the mean ratio of the non-operated (control) group ± SD (*n* = 6 independent experiments). * Significant difference from the sham-operated group (*p* < 0.05).

As GRN exerts a pro-inflammatory effect, we further investigated the mRNA levels of pro-inflammatory (TNF-α and IL-1β) and anti-inflammatory (IL-10 and TGF-β) cytokines after ME. The mRNA levels of pro-inflammatory cytokines TNF-α and IL-1β were significantly increased on day 1 after ME, compared with those of the sham-operated rats (Figure 4A,B). After that, those of these pro-inflammatory cytokines on day 7 after ME decreased to the same level seen for the sham-operated rats (Figure 4A,B). In contrast, the mRNA levels of anti-inflammatory cytokines IL-10 and TGF-β were significantly reduced on day 1 after ME as compared with those for the sham-operated rats (Figure 4C,D). Thereafter, the mRNA levels of IL-10 and TGF-β in the ME rats returned to those for the sham-operated rats (Figure 4C,D).

### 2.5. Effect of Elastase Inhibitor on Elastase Activity and on Neutrophil Infiltration after Cerebral Ischemia

**Figure 5:**
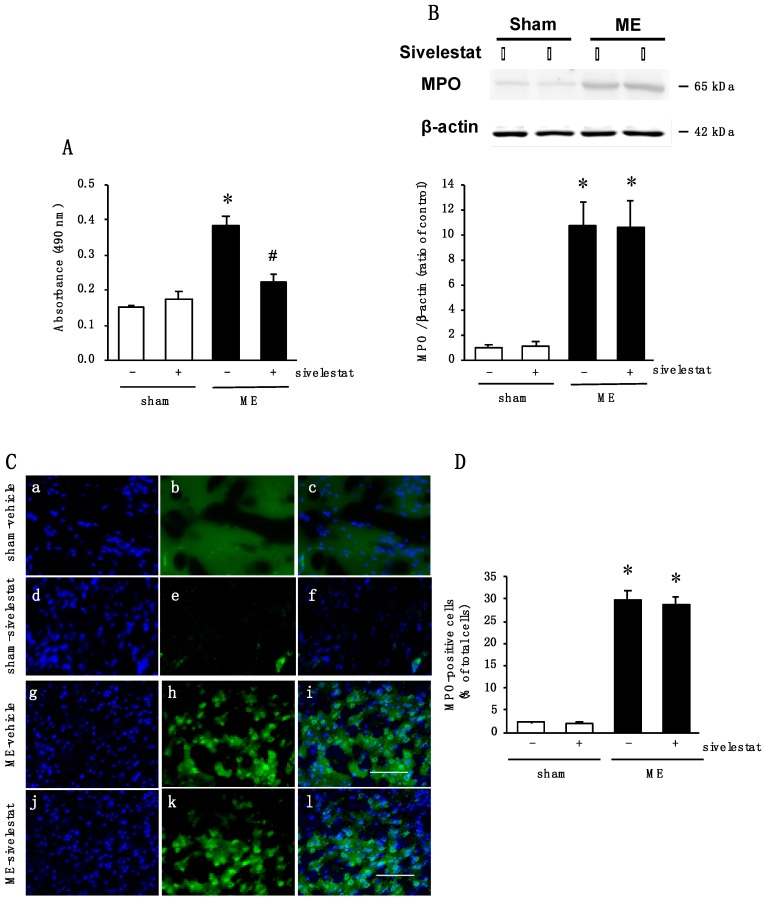
(**A**) Changes in tissue elastase activity in the vehicle-treated (-) sham and ME groups and sivelestat-treated (+) sham and ME groups on day 1 after surgery. * Significant difference from the vehicle-treated sham group (*p* < 0.05). # Significant difference from the vehicle-treated ME group (*p* < 0.05). Each value represents the mean ± SD of 6 animals; (**B**) Levels of myeloperoxidase (MPO) protein in the vehicle-treated (-) sham and ME groups and sivelestat-treated (+) sham and ME groups on day 1 after surgery. Bands corresponding to MPO were scanned, and the scanned band was normalized by β-actin on the same blot. The results are expressed as the mean ratio of the non-operated (control) group ± SD (*n* = 8 each). * Significant difference from the sham group (*p* < 0.05); (**C**) Images of double staining (merge, c, f, i, and l) with Hoechst 33342 (blue, a, d, g, and j) and MPO (green, b, e, h, and k) for the vehicle-treated sham and ME groups and sivelestat-treated sham and ME groups on day 1 after surgery. The scale bar represents 100 μm; (**D**) The number of MPO-positive cells in the vehicle-treated (-) sham and ME groups and sivelestat-treated (+) sham and ME groups on day 1 after surgery was counted. Five sections were made per animal, and 266–722 cells were counted per section, and the average of 5 sections per animal was calculated. The values for MPO-positive cells are presented as the mean ± SD (*n* = 5 each). * Significant difference from the sham group (*p* < 0.05).

We next examined the effects of an elastase inhibitor, sivelestat, on the levels of PGRN and GRN at the early stage after ME. We first confirmed that the increased activity of elastase on day 1 after ME was reduced by treatment with sivelestat (Figure 5A). In addition, the effect of administration of sivelestat on neutrophil infiltration into the brain parenchyma after ME was examined. Although, the amount of myeloperoxidase (MPO) was significantly increased after ME, the administration of sivelestat did not affect this ME-induced increase in the level of MPO (Figure 5B). Furthermore, immunohistochemical analysis indicated an increase in the number of MPO-positive cells after ME (Figure 5C,D). The increased number of MPO-positive cells was not altered by administration of sivelestat (Figure 5C,D). 

### 2.6. Effect of Elastase Inhibitor on the Levels PGRN and GRN Protein Levels and on mRNA Levels of Inflammatory Cytokines TNF-α and IL-1β after Cerebral Ischemia

**Figure 6:**
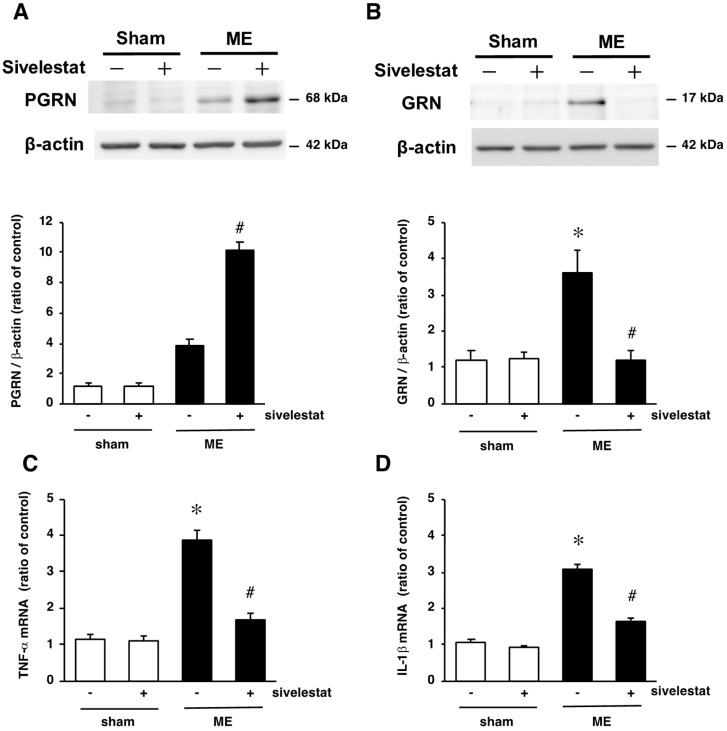
(**A**,**B**) Levels of PGRN (**A**) and GRN (**B**) protein in the vehicle-treated sham and ME groups (white bars) and sivelestat-treated sham and ME groups (black bars) on day 1 after surgery. Bands corresponding to PGRN and GRN were scanned, and the scanned bands were normalized by β-actin on the same blot. The results are expressed as the mean ratio of the non-operated (control) group ± SD (n = 8 each); (**C**,**D**) changes in the mRNA levels of TNF-α (**C**) and IL-1β (**D**) in the vehicle-treated sham and ME groups (white bars) and sivelestat-treated sham and ME groups (black bars) on day 1 after surgery. The results are expressed as the mean ratio of the non-operated (control) group ± SD (*n* = 6 each). * Significant difference from the vehicle-treated sham group (*p* < 0.05). # Significant difference from the vehicle-treated ME group (*p* < 0.05).

Next, we investigated the effects of sivelestat on PGRN and GRN proteins at the early stage after ME. The level of PGRN protein tended to increase in the vehicle-treated ME rats compared with that in the vehicle-treated sham-operated rats on day 1 after surgery (Figure 6A). The administration of sivelestat to the ME rats further increased the level of PGRN protein (Figure 6A). In contrast, there was a marked increase in the level of GRN protein on day 1 after ME was significantly attenuated by the inhibitor (Figure 6B).

Since the production of GRN was suppressed by the administration of sivelestat, we further examined the effects of sivelestat on the levels of inflammatory cytokines TNF-α and IL-1β after ME. As shown in Figure 6C,D, the increase in the levels of TNF-α and IL-1β mRNAs on day 1 after ME were inhibited by sivelestat. 

### 2.7. Effect of Elastase Inhibitor on Cell Injury and Neurological Deficits after Cerebral Ischemia

**Figure 7:**
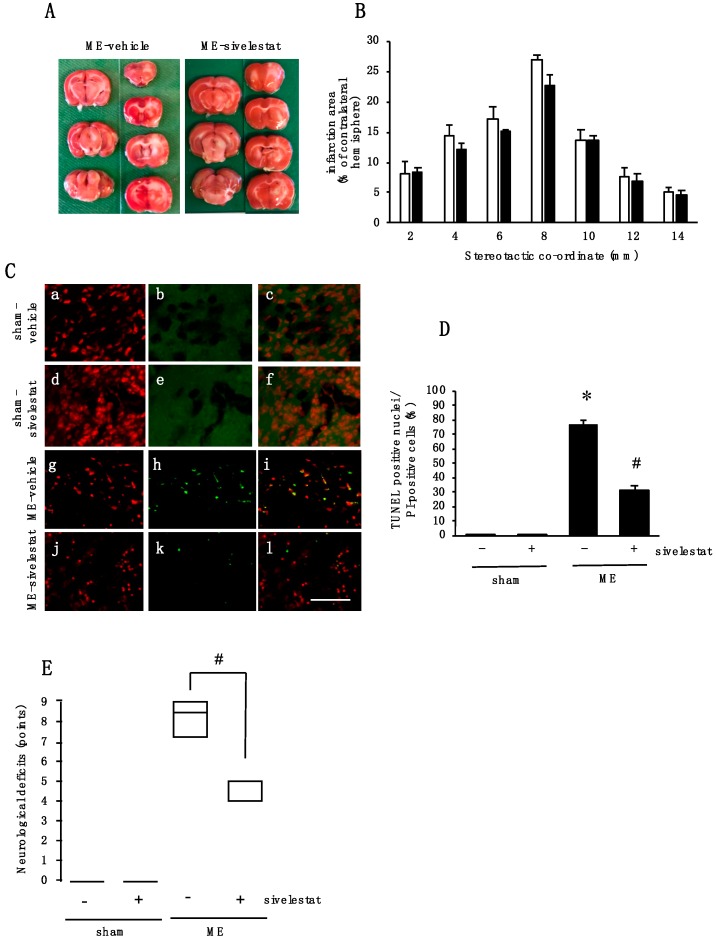
(**A**) Representative photographs of 2,3,5-triphenyltetrazolium chloride (TTC) staining from vehicle- and sivelestat-treated ME rats; (**B**) The infarct area of hemispheres at 2, 4, 6, 8, 10, 12, and 14 mm from the forebrain in the vehicle-treated ME groups (white bars) and sivelestat-treated ME groups (black bars) on day 1 after surgery. The infarct areas are expressed as percentages of the contralateral hemisphere. Values represent the means ± SD (*n* = 5 each); (**C**) images of double staining (merge, c, f, i, and l) with PI (red, a, d, g, and j) and TUNEL-positive nuclei (green, b, e, h, and k) for the vehicle-treated sham, ME groups, sivelestat-treated sham, and ME groups on day 1 after surgery. The scale bar represents 50 μm; (**D**) Effect of sivelestat administration on the number of TUNEL-positive nuclei in the vehicle-treated (-) sham and ME groups and sivelestat-treated (+) sham and ME groups on day 1 after surgery. The number of TUNEL-positive nuclei in the cerebral cortex was counted. Five sections were made per animal, and 319 to 1194 cells were counted per section, and the average of 5 sections per animal was calculated. Values represent the means ± SD (*n* = 5 each); (**E**) changes in the neurological deficits of vehicle-treated (-) sham and ME groups and sivelestat-treated (+) sham and ME groups. Each value represents medians and interquartile (*n* = 8 each). # Significant difference from the vehicle-treated ME group (*p* < 0.05).

Furthermore, we examined the effects of sivelestat administration on cell injury after ME. Although, there was no effect on infarct areas (Figure 7A,B), terminal deoxynucleotidyl transferase-mediated dUTP-biotin nick-end labeling (TUNEL)-positive nuclei were evident on day 1 after ME (Figure 7C,D). The administration of sivelestat almost completely abolished the expression of TUNEL-positive nuclei induced by ME (Figure 7C,D). Finally, we examined the effect of sivelestat treatment on ME-induced neurological deficits. Sivelestat significantly decreased the score of neurological deficits on day 1 after ME (Figure 7E). Sham-operated rats did not reveal any neurological deficits with or without treatment of sivelestat treatment (Figure 7E). 

## 3. Discussion

In this study, we examined the pathophysiological changes in PGRN and GRN in the cerebral cortex after ME. Moreover, the effects of the neutrophil elastase inhibitor sivelestat on PGRN cleavage and GRN production were investigated.

We demonstrated that the level of PGRN protein in the cerebral cortex was significantly increased after ME. Furthermore, PGRN was expressed in the microglia of the ipsilateral hemisphere after ME. Microglia play important roles in various types of brain injury and disease, including cerebral ischemia and Alzheimer’s disease [17]. Microglia are normally dispersed throughout the brain parenchyma with a highly ramified form. In addition, in vivo two-photon imaging revealed that microglia are dynamic, extending and retracting their processes in a random manner and interspersed with brief periods [18]. The inflammatory response, induced by ischemic brain injury, involves the activation of resting microglia in the central nervous system [19], and it is known that microglia is activated by cerebral ischemia accumulate at the site of injury [20]. Therefore, the increase in PGRN after ME seen in the present study might be attributed to the accumulation of microglia. Once activated, microglia are able to modify their morphology and function by switching between M1 and M2 phenotypes [21]. Those with the M1 polarized phenotype secrete pro-inflammatory cytokines, such as IL-6, IL-1β, and TNF-α, which may further aggravate neuroinflammation in the peri-infarct region after ischemia and reperfusion injury [22]. In this regard, we demonstrated that round and ameboid-like microglia were evident after ME, and that the mRNA levels of pro-inflammatory cytokine (TNF-α and IL-1β) were significantly increased in cerebral cortex after ME, as well as in cultured microglia after OGD treatment. These findings suggested that microglia after ME and OGD treatment, conducted in this study, would be of the M1 phenotype. Furthermore, as the levels of PGRN mRNA were also elevated in microglia after OGD treatment, the major cellular source of PGRN may be considered to be M1 microglia. Although, the clarification of the relationship between the expression of PGRN mRNA and pro-inflammatory cytokine mRNA is needed, our findings raise the possibility that the increase in the PGRN mRNA level in microglia plays a key role in anti-inflammatory responses in the acute phase under the cerebral ischemia-induced pathophysiological condition.

It has been reported that PGRN plays anti-inflammatory roles, but that GRN cleaved from PGRN plays the inverse ones [9]. While the increase in the level of anti-inflammatory factor, PGRN, was evident, elastase activity increased at an early phase after ME. This increased activity of elastase after ME might be related to production of GRN, which causes inflammation. Neutrophils are transiently detected in the brain parenchyma after ischemia, since they are rapidly attracted to the injured brain at an early stage after ischemia [23]. Neutrophil elastase, a member of the chymotrypsin superfamily of serine proteases, is a 22-kDa enzyme, which is rapidly released from the azurophilic granules into the extracellular space in inflamed tissues [24]. During an inflammatory reaction, neutrophils secrete an elastase that cleaves of PGRN into GRN peptide, potentially exacerbating the inflammation [9]. These findings suggest that an increase in intracerebral neutrophil elastase activity at the early stage of cerebral ischemia, led to the cleavage of PGRN, causing a GRN-induced inflammatory response. Therefore, neutrophils also contribute to tissue damage after ischemia [25]. Other studies reported that the administration of r-PGRN significantly attenuated neuronal injury induced by cerebral ischemia-reperfusion [26]. Furthermore, transgenic mice over-expressing PGRN had reductions in their cerebral infarct area and had better functional outcomes after focal cerebral ischemia than did wild-type mice [27]. These findings suggest that inhibition of neutrophil elastase-induced PGRN cleavage by the elastase inhibitor may possibly suppress the development of cerebral infarction. Alternatively, GRN produced by neutrophil elastase-induced cleavage of PGRN has the ability to exacerbate the inflammatory responses after cerebral ischemia.

Sivelestat is a specific neutrophil elastase inhibitor [28] and has been reported to attenuate pulmonary inflammation and fibrosis in animal models [29,30]. Its effects on lung and cardiovascular diseases have been examined in several clinical trials [31,32]. In this study, we demonstrated that the administration of sivelestat inhibited the cleavage of PGRN and the production of GRN after ME. Sivelestat inhibits the activity of neutrophil elastase during the acute inflammatory phase of monoiodoacetate-induced experimental osteoarthritis model. Moreover, sivelestat decreases joint edema and reduces the number of rolling and adherent leukocytes, suggesting that neutrophil elastase contributed to leukocyte extravasation in the early inflammatory phase of monoiodoacetate-induced osteoarthritis [33]. Furthermore, sivelestat suppresses pro-inflammatory cytokines (TNF-α and IL-6) in liver ischemia-reperfusion injury in animal models [34]. However, the mechanisms underlying the effect of sivelestat on inflammatory responses are still incompletely understood. Our data suggest that suppression of PGRN cleavage and GRN production by sivelestat administration could be a potential therapeutic strategy for inflammatory responses after cerebral ischemia. Because the levels of MPO in ME rats were not affected, regardless of administration with sivelestat, the effects of sivelestat on the levels of PRGN and GRN were not dependent on neutrophil infiltration. In addition, the administration of sivelestat decreased the mRNA levels of TNF-α and IL-1β, without changes in the infiltration of neutrophils in the present study. These results suggest that sivelestat might have decreased the expression of pro-inflammatory cytokines caused by GRN. It was reported that intracerebroventricular injection of recombinant PGRN enhanced recovery of the neurological function after transient focal cerebral ischemia [26], possibly through the neuroprotective and anti-inflammatory effects of PGRN and induction of growth factor. In this sense, we demonstrated that the administration of sivelestat decreased the number of TUNEL-positive nuclei and improved the score of neurological deficits. Although, the mechanisms by which sivelestat-induced functional recovery after ME have not yet been fully elucidated. Our findings suggest that the anti-inflammatory effects of sivelestat play multiple essential roles under pathophysiological conditions, including cerebral ischemia.

## 4. Materials and Methods

### 4.1. Model of Microsphere-Induced Cerebral Embolism in Rats

Male Wistar rats weighing between 220 and 250 g (Charles River Japan Inc., Tsukuba, Japan) were used in the present study. The rats were maintained at 23 ± 1 °C in a room with a constant humidity of 55 ± 5% and a light cycle of 12-h light/12-h darkness and had free access to food and water according to the National Institute of Health Guide for the Care and Use of Laboratory Animals and the Guidance for Experimental Animal Care issued by the Prime Minister’s Office of Japan. The study was approved by the Committee of Animal Care and Welfare of Tokyo University of Pharmacy and Life Sciences (P18-62, 19 April 2018).

Microsphere-induced cerebral embolism (ME) was performed by the method described previously [35]. The rats were anesthetized by 5% isoflurane and maintained with 2.5% isoflurane, after which their right external carotid and pterygopalatine arteries were temporarily occluded with strings. Immediately thereafter, a needle connected to a polyethylene catheter (TORAY Feeding Tube, Chiba, Japan) was inserted into the right common carotid artery; and then 700 microspheres (45.0 μm in diameter; Polysciences Inc., Warrington, PA, USA), suspended in 20% dextran solution, were injected into the right internal carotid artery through the cannula. After the injection (150 µL), the needle was removed, and the puncture wound was then repaired with surgical glue. The rats that underwent a sham operation received the same volume of vehicle without microspheres. Non-operated rats were used as naïve control rats in the present study.

### 4.2. Assessment of Neurological Deficit

On day 1 after surgery, neurological deficits of the operated rats were assessed on the basis of paucity of movement, truncal curvature, and forced circling during locomotion, according to the criteria described previously [36,37,38]. The neurological deficit was scored by a researcher who was blinded to the experimental groups using the following scale (3, very severe; 2, severe; 1, moderate; 0, little or none) for each of the three symptoms (paucity of movement, truncal curvature, and forced circling during locomotion). The results were expressed as the total score (maximal total score, 9) of the three symptoms.

### 4.3. Drug Administration

Siverestat (Nipro, Osaka, Japan), which is a selective inhibitor of neutrophil elastase, was dissolved in phosphate-buffered saline (PBS; 137 mM NaCl, 8.1 mM Na_2_HPO_4_, 2.7 mM KCl, 1.5 mM KH_2_PO_4_, pH 7.4) and administered (50 mg/kg) intravenously twice, just after the surgery for ME and then once subcutaneously 8 h after ME. Administration of sivelestat immediately after ME aimed to reduce neutrophil elastase activity in blood and to inhibit elastase activity of neutrophils, which has infiltrated into brain parenchyma by ischemia-induced BBB breakdown. The administration after 8 hours (second time) aimed to maintain the blood concentration of sivelestat. The dose of sivelestat and this type of drug administration were based on the reports of Ikegama et al. [39] and Tonai et al. [40].

### 4.4. Isolation and Culture of Cortical Microglia

The rats were maintained according to the National Institute of Health Guide for the Care and Use of Laboratory Animals and the Guideline for Experimental Animal Care issued by the Prime Minister’s Office of Japan. All experimental procedures were approved by the Committee of Animal Care and Welfare of Tokyo University of Pharmacy and Life Sciences.

Mixed glial cultures were prepared from cerebral cortices of Wistar rats on postnatal day 3 (SLC, Shizuoka, Japan), according to the method of Giulian and Baker [41], with minor modifications. The cortices were minced and digested by incubation for 20 min at 37 °C in PBS, containing 0.25% trypsin (Invitrogen, Carlsbad, CA, USA) and 1 mg/mL DNase I (Worthington Biochemical Co., Lakewood, NJ, USA). After trituration by using a fire-polished Pasteur pipet in DMEM/Ham’s F12 medium (Thermo Fisher Scientific, Waltham, MA, USA) containing 20% heat-inactivated fetal bovine serum (FBS) and 1% penicillin-streptomycin (Wako, Osaka, Japan), the isolated cells were pelleted by centrifugation at 120× *g* and then resuspended in fresh DMEM/F12 medium containing 10% FBS. Next, these cells were plated at a density of 500,000 cells/well in 24-well plates (Falcon, Corning, NY, USA) or as 1 cortex/75 cm^2^ flask (Falcon) coated with poly-D-lysine (Wako). The cultures were maintained at 37 °C and 5% CO_2_ in DMEM containing 10% FBS. One half of the medium was replaced with fresh medium every 3 days. Rat microglial cells were harvested from mixed glial cell primary cultures prepared from neonatal rats, as previously reported [42]. Confluent cultures of mixed glia cells were maintained in flasks for 3 weeks, and then incubated at 37 °C with DMEM/F12 containing 0.0625% trypsin for 30 min. The astrocyte layer was lifted by this treatment, whereas the microglial cells remained attached to the bottom of the well. The specificity and purity of the cultured microglial cells was confirmed by immunostaining with Iba-1.

### 4.5. Western Immunoblotting

On days 1, 3, or 7 after surgery, ME- or sham-operated rats were decapitated. The right hemisphere was homogenized in ice-cold buffer containing 10% sucrose, 1 mM ethylenediaminetetraacetic acid (EDTA), and protease inhibitor cocktail (Roche Diagnostics GmbH, Germany) in 20 mM Tris–HCl (pH 7.4). Then the protein concentration was determined. Samples were heated at 95 °C for 5 min in 10% glycerol and 2% sodium dodecyl sulfate (SDS) in 62.5 mM Tris-HCl (pH 6.8). Western blotting was performed according to standard protocols. The following primary antibodies were used: mouse anti-β-actin (dilution, 1:10,000; a1978, Sigma), rabbit anti-PGRN (dilution, 1:1000; 18410-1-AP, Proteintech, Tokyo, Japan), which also recognize GRN, and rabbit anti-myeloperoxidase (dilution, 1:1000; MPO, ab65871, Abcam, Cambridge, UK) antibodies. Quantification was performed by using computerized densitometry (Luminograph II, ATTO Co., Tokyo, Japan) and an image analyzer (CS Analyzer, ATTO Co., Tokyo, Japan).

### 4.6. qRT-PCR

The total RNA was extracted from the right hemisphere of rats and from primary cultures of cortical microglia by using an RNA extraction kit, Isogen II (Nippon Gene, Tokyo, Japan) and quantified by BioSpec-nano (Shimazu Corp., Kyoto, Japan). cDNAs were synthesized from 500 ng of total RNAs by using ReverTra Ace^®^ qPCR RT Master Mix with gDNA Remover (TOYOBO CO., LTD., Tokyo, Japan). qRT-PCR was performed using THUNDERBIRD^®^ SYBR qPCR Mix (TOYOBO CO., LTD.) on a CFX Connect Real-Time PCR Detection System (Bio-Rad Laboratories, Hercules, CA, USA). Data were normalized to the 18S rRNA mRNA expression and analyzed by the 2^−ΔΔCt^ method. Primers used in the present study were as follow: 18S rRNA—forward, 5′-CGGACAGGATTGACAGATTG-3′; reverse, 5′-CAAATCGCTCCACCAACTAA-3′. PGRN—forward, 5′-CGGACAGGATTGACAGATTG-3′; reverse, 5′-CAAATCGCTCCACCAACTAA-3′. IL-1β—forward, 5′-AGCTGCACTGCAGGCTTCGAGATG-3′; reverse, 5′-GAACTGTGCAGACTCAAACTCCAC -3′. IL-10 —forward, 5′- AGTGGAGCAGGTGAAGAATGA-3′; reverse, 5′-TCATGGCCTTGTAGACACCTT-3′. TNF-α—forward, 5′-ACCACGCTCTTCTGTCTACTG-3′; reverse, 5′-CTTGGTGGTTTGCTACGAC-3′. TGF-β—forward, 5′-GTCAACTGTGGAGCAACACG-3′; reverse, 5′-AGACAGCCACTCAGGCGTAT-3′.

### 4.7. Immunohistochemistry

On day 3 after surgery, ME- and sham-operated rats were perfused via the heart with 4% paraformaldehyde in 0.1 mol/L phosphate buffer. Their brains were then quickly removed, and immersed in 30% sucrose in 0.1 mol/L phosphate buffer, and subsequently cut into 5-mm-thick coronal slabs. The latter were subsequently embedded in Neg50 (Richard-Allan Scientific, Kalamazoo, MI, USA) and cut into 10-µm sections by using a cryostat. For immunostaining, the following primary antibodies were used: Goat monoclonal anti-Iba-1 (dilution, 1:200; ab5076, Abcam), rabbit polyclonal anti-PGRN (dilution, 1:100; 18410-1-AP, Proteintech, Tokyo, Japan), and rabbit anti-MPO (dilution, 1:100; ab65871, Abcam) antibodies. The secondary antibodies used were the following: Alexa Fluor 594-labeled donkey anti-goat IgG (dilution, 1:200; A11058; Invitrogen), Alexa Fluor 488-labeled donkey anti-rabbit IgG (dilution, 1:200; A21206; Invitrogen) and Alexa Fluor 488-labeled goat anti-rabbit IgG antibodies (dilution, 1:200; A11008; Invitrogen) antibodies, respectively. The nuclei were stained with Hoechst33342 (dilution, 1:2000; 346-07951, Dojindo, Kumamoto, Japan). Fluorescence was detected by using an Olympus fluorescence microscope (IX-71; Olympus, Tokyo, Japan). The omission of primary antibodies served as a negative control. No immunostaining was detected in this group. Fluorescent images were loaded into the MetaMorph software program (Molecular Devices, Downingtown, PA, USA). Based on the background fluorescence and the size of their nucleus, the antibody-labeled cells of the cerebral cortex were observed using the MetaMorph software program (5 sections per animal), where the areas corresponded to coronal coordinates of −2.3 to 0.70 from bregma.

### 4.8. 2, 3, 5-Triphenyltetrazolium Chloride Staining

The infarct size after ME was evaluated by 2,3,5-triphenyltetrazolium chloride (TTC)-staining of brain slices. In brief, coronal sections with a 2-mm width were made on day 1 after ME, and the slices were incubated with 2% TTC in physiological saline. TTC-unstained areas were analyzed by use of an image analyzer (NIH image 1.63, NIH, Bethesda, MD, USA).

### 4.9. Terminal Deoxynucleotidyl Transferase-Mediated dUTP-Biotin Nick end Labeling

Terminal deoxynucleotidyl transferase-mediated dUTP-biotin nick end labeling (TUNEL)-positive nuclei were detected by using an in situ Apoptosis Detection Kit (MK500; Takara Bio Inc., Shiga, Japan). Surviving cells and TUNEL-positive nuclei in the cerebral cortex were counted under ×400 magnification (IX-71; Olympus) in 5 sections per animal, which corresponded to coronal coordinates of −2.3 to 0.70 from bregma. Images (657.34 × 863.41 µm) were assessed by quantifying the number of stained cells as a percentage of total number of cells with the Multi Wavelength Cell Scoring Module of MetaMorph software (Molecular Devices). 

### 4.10. Elastase Activity

On days 1, 3, and 7 after surgery, ME- or sham-operated rats were decapitated and their brains immediately removed and flash-frozen. Tissue samples were homogenized in extraction buffer containing 50 mM Tris-HCl (pH 7.6), 150 mM NaCl, 5 mM CaCl_2_, and 0.05% Brij-35. Total protein concentrations were determined by using a Pierce^TM^ BCA Protein Assay (Thermo Fisher Scientific), as per the manufacturer’s instructions. Equal amounts of protein were incubated with 10 mg/mL Elastin-Congo Red (E164; Elastin Product, Owensville, MO, USA) at 37 °C during rotation for 16 h. The solution was centrifuged to pellet any undigested and insoluble elastin. The absorbance of elastin-Congo red cleaved by the elastolytic activity was measured at 490 nm.

### 4.11. Oxygen and Glucose Deprivation (OGD)

For cultures in an OGD environment, the cells were placed in an OGD chamber, which was initially flushed with a mixture of 95% N_2_ and 5% CO_2_, within a humidified modular incubator 37 °C. The oxygen concentration in the chamber was maintained at 5% with a residual gas mixture of 5% CO_2_ and balanced nitrogen for 24 h at 37 °C. The original culture medium was changed to DEME media without glucose (Thermo Fisher Scientific). For cultures in the normoxic environment, cells in a matched control group were cultured in 95% atmospheric air and 5% CO_2_ for the same times as cells under the OGD condition.

### 4.12. Immunocytochemistry

Microglia were fixed with 4% paraformaldehyde and blocked with 10% goat serum and 1% bovine serum albumin in Triton X-100 in PBS. The primary antibodies used were goat monoclonal anti-Iba-1 (dilution, 1:200; ab5076, Abcam), rabbit polyclonal anti-GFAP (dilution, 1:200; ab7260, Abcam), and rabbit polyclonal anti-PGRN (dilution, 1:100; 18410-1-AP, Proteintech) antibodies. The secondary antibodies used were Alexa Fluor 594-labeled donkey anti-goat IgG (dilution, 1:200; A11058; Invitrogen) and Alexa Fluor 488-labeled donkey anti-rabbit IgG (dilution, 1:200; A21206; Invitrogen) antibodies, respectively. Nuclei were stained with Hoechst33342 (dilution, 1:2000; 346-07951, Dojindo, Kumamoto, Japan). Fluorescence was detected by using an Olympus fluorescence microscope (IX-71; Olympus). Fluorescent images were loaded into the MetaMorph software program (Molecular Devices, Sunnyvale, CA, USA). Five images (435 × 330 μm) per experiment were randomly taken from 9 independent experiments and were analyzed with the MetaMorph software program (Molecular Devices).

### 4.13. Statistical Analysis

Statistical analyses were performed with GraphPad Prism (version 8, GraphPad Software, San Diego, CA, USA). To define whether the data were normally distributed, one sample Kolmogorov-Smirnov test was used. For the normally distributed data, the differences between the 2 groups were evaluated statistically by use of the unpaired Student’s t test, and differences among multiple groups were performed by using factorial analysis of variance (ANOVA), followed by the Tukey test as a post hoc test. The statistical analyses for neurological deficit scores, which were not normally distributed, was performed using the Kruskal-Wallis analysis, and significant differences between the groups were determined by a non-parametric Wilcoxon-Mann-Whitney test. The results were expressed as the means ± standard deviation (SD) and except for the neurological deficit scores, which were expressed as medians and interquartile ranges. P values of less than 0.05 were considered to indicate statistical significance.

## 5. Conclusions

Our findings suggest that the observed increase in intracerebral neutrophil elastase activity in the early phase of cerebral ischemia may cause an inflammatory response by cleavage of PGRN to produce GRN. Moreover, inhibition of neutrophil elastase activity, after ME, by the administration of sivelestat suppressed PGRN cleavage and GRN production. These results demonstrated that the suppression of PGRN- and GRN-related inflammatory responses, after cerebral ischemia, by the administration of sivelestat could suppress the progression of ischemic injury. Our results regarding the administration of sivelestat after cerebral ischemia provide new insight into the pathophysiology of this ischemia, and we expect that the increase in levels of PGRN, and also the decrease in the levels of GRN, elicited by sivelestat, will lead to the establishment of a new treatment for acute cerebral ischemia.

## Abbreviations

PGRN	Progranulin
GRN	Granulin
t-PA	Tissue plasminogen activator
BBB	blood-brain barrier
ME	Microsphere-induced cerebral embolism
OGD	Oxygen/glucose deprivation
GFAP	glial fibrillary acidic protein
MPO	Myeloperoxidase
TUNEL	Terminal deoxynucleotidyl transferase-mediated dUTP-biotin nick end labeling
